# Severe COVID-19 among Admitted COVID-19 Patients in a Tertiary Care Centre: A Descriptive Cross-sectional Study

**DOI:** 10.31729/jnma.8074

**Published:** 2023-04-30

**Authors:** Sapana Sedhain, Amrita Sinha, Neeta Kafle, Dipak Kumar Thakur

**Affiliations:** 1 Department of Pathology, Birat Medical College Teaching Hospital, Biratnagar, Morang, Nepal; 2 Department of Urology, Birat Medical College Teaching Hospital, Biratnagar, Morang, Nepal

**Keywords:** *COVID-19*, *c-reactive protein*, *lymphocytes*, *severe acute respiratory syndrome coronavirus*

## Abstract

**Introduction:**

Severe COVID-19 patients experience elevated levels of serologic indicators of inflammation which can alter blood cell lineages and cause lymphopenia. The objective of this study was to find out the prevalence of severe COVID-19 among admitted COVID-19 patients in a tertiary care centre.

**Methods:**

A descriptive cross-sectional study was conducted in a tertiary care centre from 22 June 2021 to 30 September 2021 after obtaining ethical approval from the Institutional Review Committee (Reference number: IRC-PA-146/2077-78). Confirmed COVID-19 patients by reverse transcriptase polymerase chain reaction and admitted in the COVID block during the study period were included and those who were discharged on request or referred or unavailable blood tests were excluded. A convenience sampling method was used. Point estimate and 95% Confidence Interval were calculated.

**Results:**

Among 72 admitted COVID-19 patients, 63 (87.5%) (79.86-95.14, 95% Confidence Interval) patients had severe disease. The mean neutrophil to lymphocyte ratio and mean lymphocyte to C-reactive protein ratio were 11.60±8.15 and 25.55±20.96 respectively.

**Conclusions:**

The prevalence of severe COVID-19 was higher than in other studies done in similar settings. We suggest clinical parameter-based early categorisation of COVID-19 cases to utilize limited resources during the pandemic.

## INTRODUCTION

During the pandemic of COVID-19, categorising patients into severity groups allows proper utilisation of limited resources and a reduction in disease-related morbidity and mortality.^1^ COVID-19 patients experience elevated levels of markers of inflammation, like C-reactive protein (CRP), erythrocyte sedimentation rate (ESR), lactate dehydrogenase (LDH), and procalcitonin.^2,3^

They have a higher level of inflammatory cytokines altering the levels of various blood cell lineages causing lymphocytopenia. These cytokines stimulate several downstream pathways, increasing the production of acute-phase reactants like CRP, and increasing the mobilization of neutrophils. This, along with stress-induced neutrophilia, likely explains the relative lymphopenia.^4^ Thus, biomarkers like neutrophil-to-lymphocyte ratio (NLR) and lymphocyte-to-CRP ratio (LCR) can be used in predicting the severity of COVID-19.

The objective of this study was to find out the prevalence of severe COVID-19 among admitted COVID-19 patients in a tertiary care centre.

## METHODS

This was a descriptive cross-sectional study conducted in the Birat Medical College Teaching Hospital, Morang, Nepal during the second wave of COVID-19 from 22 June 2021 to 30 September 2021. Ethical approval was obtained from the Institutional Review Committee of the same hospital (Reference number: IRC-PA-146/2077-78). Those cases who were confirmed COVID-19 positive by reverse transcriptase polymerase chain reaction and who were admitted in the COVID block of the same hospital during the study period were included. Those cases who were discharged on request or referred or whose blood test was not available were excluded. A convenience sampling method was used. The sample size was calculated using the following formula:


$$\begin{array}{ll} \text{n} & ={\text{Z}}^{2}\times \frac{\text{p}\times \text{q}}{{\text{e}}^{\text{2}}}\\ & =1.96^{2}\times \frac{0.20\times 0.80}{0.1^{2}}\\ & =62 \end{array}$$

Where,

n = minimum required sample size
Z = 1.96 at 95% Confidence Interval (CI)
p = prevalence taken as 20% severe COVID-19 in a similar study^5^
q = 1-p
e = margin of error, 10%

The minimum sample size calculated was 62. However, a total of 72 patients were included in the study.

At admission, patient demography was recorded and categorized into severe and non-severe COVID-19 based on clinical parameters (for severe disease: respiratory rate >30/min or SpO2 <93% or PaO2/FiO2 ≤300 mmHg or presence of >50% lung infiltrates or shock or mechanical ventilation implementation or intensive care admission).^1^

Baseline laboratory parameters were sent including complete blood count and CRP. Venous blood samples were collected through venipuncture from COVID-19 patients into tubes coated with gel for serum separation. The blood sample was centrifuged at 4000 rpm for 5 minutes within 1 hour. Serum CRP levels (cut-off value 1 mg/l) were determined by semiautomated Mispa-i2 specific protein analyzer Nephelometer. Whole blood samples were collected into ethylenediaminetetraacetic acid (EDTA) containing tubes. Total white blood cell (WBC) count, Lymphocyte and Neutrophil count were measured using Beckman Coulter DxH Hematology Analyzer. The NLR was calculated by dividing the absolute neutrophil count by the absolute lymphocyte count. The LCR was calculated by dividing absolute lymphocyte count by CRP (mg/dl).

The information was recorded on predesigned proforma and analysed using IBM SPSS Statistics 27.0. Point estimate and 95% CI were calculated.

## RESULTS

Among 72 admitted COVID-19 patients, 63 (87.5%) (79.86-95.14, 95% CI) patients had severe disease. A total of 32 (50.80%) were male and 31 (49.20%) were females. The mean age of the severe COVID-19 patients was 54.98±17.80 years. The mean NLR and LCR in severe patients were 11.60±8.16 and 25.34±21.04 respectively (Table 1).

**Table 1:** Baseline characteristics of severe COVID-19 patients (n= 63).

Parameter	Mean±SD
Age (years)	54.98±17.80
WBC (cells/mm^3^)	19030±8827
Neutrophils (10^9^/L)	87.03±11.86
Lymphocytes (10^9^/L)	12.62±11.53
CRP (mg/dL)	102.05±92.37
NLR	11.60±8.16
LCR (10^9^/l/mg/dl)	25.34±21.04

## DISCUSSION

On the basis of clinical features, 63 (87.5%) patients were categorized into severe COVID-19 in this study. A similar study done in Turkey showed (60.65%)^6^ severe COVID-19 while in another study done in China, it was 35%.^7^ In contrast, One study done in Iran showed severe cases in 20% only.^5^ Whereas in another study done in the initial phase of the COVID-19 outbreak in China, the severe disease was seen in 15.74% of cases only (173/1099).^8^ Similarly, In another study done in China, the prevalence of severe disease was 5.62% only (5/89).^9^ These differences in the prevalence of severe COVID-19 can be explained by the following facts. Our study was conducted during the second wave of COVID-19 when the pandemic had the worst course affecting more people severely. This can also be explained by the fact that during the second wave, most of the patients with mild symptoms remained in home isolation and treatment while only severe cases came to the hospital. These facts could be the reasons for the higher number of severe cases in the study.

In this study, males and females were equally (51% vs 49%) affected indicating no gender is spared from containing COVID-19 and the mean age was 54.98 years. Our results are comparable with the results of a similar study done in Iran. Here males were 57% and females were 43% (n= 70) and the mean age was 42.7 years.^5^ Similar results were also noted in the study done in Turkey where 59% of males and 41% of females were involved with a mean age of 58.55 years.^6^

The mean NLR found in our study for severe COVID-19 patients was 11.60 (6.51 in non-severe). This is comparable to a similar study done in India where NLR was more markedly raised in a severe group (10.8 vs 7.36).^10^ Other studies also concluded that the higher the NLR, severe the COVID-19 and mortality.^5,11,12^

When looking at the status of LCR in the severe group, it was markedly decreased (25.55 vs 62.23) indicating rising CRP and lymphopenia with the severity of COVID-19. A similar result was also demonstrated in a study done in Turkey, decreasing LCR with the disease severity.^6^ This is similar to the result of a meta-analysis done in Mexico demonstrating that the LCR was decreased in severe cases.^13^ These lower LCR levels in severe patients could be the result of fewer lymphocytes leading to immune dysfunction and higher CRP levels reflecting the severe systemic inflammatory response of the patients.

Considering the sampling method used in this study, the chances of selection bias are there. This study is further limited by including the admitted patients only and being a single-centre study so this result may not represent the true prevalence of the condition in other settings. We recommend further multicentric studies with large sample sizes.

## CONCLUSIONS

The prevalence of severe COVID-19 in our setting was higher than in other studies done in similar settings. We suggest clinical parameter-based early categorisation of COVID-19 cases to utilise limited resources in the pandemic.
